# Genotype–phenotype correlation in migraine without aura focusing on the rs1835740 variant on 8q22.1

**DOI:** 10.1007/s10194-011-0386-5

**Published:** 2011-10-01

**Authors:** Anne Francke Christensen, Han Le, Malene Kirchmann, Jes Olesen

**Affiliations:** Department of Neurology, Danish Headache Center, University of Copenhagen, Glostrup University Hospital, Nordre Ringvej 57, Glostrup, 2600 Copenhagen, Denmark

**Keywords:** Migraine, Migraine without aura, Phenotype, Genotype, Clinical characteristics

## Abstract

A large two-stage GWAS by Antilla et al. reported the minor allele of rs1835740 on 8q22.1 to be associated with common types of migraine. The objective of the present study was to determine the clinical correlate of the variant in migraine without aura (MO). Clinical data on 339 successfully genotyped MO patients (patients with attacks of migraine without aura and no attacks of migraine with aura) were obtained by an extensive validated semi-structured telephone interview performed by a physician or a trained senior medical student. Reliable, systematic and extensive data on symptoms, age of onset, attack frequencies and duration, relevant comorbidity, specific provoking factors including different hormonal factors in females, and effect and use of medication, both abortive and prophylactic, were thereby obtained. A comparison of carriers and non-carriers were performed. Comparison of homozygotes with heterozygotes was not performed as the number of homozygotes was too small for statistical purposes. Data from other MO populations in the GWAS by Antilla et al. were not included as phenotype and clinical data were obtained differently. While thousands of patients are needed to detect a genetic variant like rs1835740, 339 are sufficient to detect meaningful clinical differences. 136 of 339 patients were carriers of the variant, 15 were homozygous. Comparison of carriers with non-carriers showed no significant difference in any of the parameters studied. In conclusion, the rs1835740 variant has no significant influence on the clinical expression of MO.

## Introduction

Genetic factors have been demonstrated to play an important role in the pathogenesis of migraine. The rare familial hemiplegic migraine (FHM), defined by the presence of a transient hemiplegia during the aura phase, is dominantly inherited with at least four different genetic subtypes [1]. In contrast, migraine without aura (MO) and migraine with typical aura (MA) have multifactorial inheritance [2–7]. The co-occurrence of MO and MA in monozygotic and dizygotic twin pairs is not higher than expected by chance, which together with the different epidemiology and clinical features of the two indicates that MO and MA are distinct disorders with some clinical and etiological overlap [5, 8]. This study will concentrate on MO. The influence of a genetic factor in MO is supported by a twofold increased risk of MO in first degree relatives of probands with MO compared to the general population [3]. Furthermore, the concordance rate of MO in monozygotic twin pairs is significantly higher than in dizygotic twin pairs (28 vs. 18%) [9], and the heritability has been estimated to 50% [2].

Until recently, no reproducible genetic association or linkage has been reported in MO. However, a large two stage genome-wide association study (GWAS) by Antilla et al. reported the minor allele of rs1835740 on chromosome 8q22.1 to be associated with migraine with an overall meta-analysis *p* value of 1.69 × 10^−11^ [10]. The study comprised case materials from five European countries, 2,731 migraine cases were included in the discovery stage and 3,202 in the replication stage. The allele was found to be overrepresented in all of the following groups: all migraine patients, MA patients without attacks of MO (MA only), patients with attacks of both MA and MO, and MO patients without attacks of MA (MO only).

The clinical correlate of this variant, if any, remains to be determined. In our genetic studies, we have systematically collected extensive clinical data using a semi-structured telephone interview by a physician. We are thus able to report on the phenotype–genotype correlation based on detailed clinical information in 339 patients with MO and no attacks of MA from the Danish subsample. The aim of the present study was thus to compare clinical data in MO patients with and without the rs1835740 variant.

We have not attempted to include the other groups from the two-stage GWAS in this analysis in that the phenotyping has been done differently and with different elaboration on clinical features from group to group. Thus, it was not possible to pool clinical data. If the difference in clinical features between patients with and without the variant is of any clinical importance, 339 patients should be enough for this purpose.

## Methods

### Ascertainment of MO patients without attacks of MA (MO only)

Patients having a MO diagnosis living in the eastern part of Denmark were extracted from case files at the Danish Headache Center. The recruited patients, all ethnical Caucasians, received a posted letter stating that the objective of the survey was to study the inheritance of migraine and would involve a telephone interview and a blood sample. They were asked to return a slip in a prepaid envelope confirming whether they agreed to participate. 606 patients were recruited and 407 agreed to participate, corresponding to a response rate of 67.4%. These patients were then contacted for an extensive semi-structured telephone interview by a physician or a trained senior medical student. The validated semi-structured telephone interview was based on the diagnostic criteria of The International Headache Society (IHS), and the diagnosis was given according to the IHS 2004 criteria [11, 12]. A total of 360 patients were given the diagnosis MO. Of these, 56 were excluded because of co-occurrence of MA. Thus, 304 were given the diagnosis ‘MO only’ and included. In addition, 81 MO-only patients were diagnosed and included from another study recruiting MA families using the same semi-structured telephone interview [13]. The total number of MO-only patients was thus 385. A blood sample was collected from each of the 385 MO-only patients, and 340 were successfully genotyped. One was hereafter excluded due to missing data; hence, the analysis in this study was based on data in 339 MO-only patients. All subjects provided written informed consent. The project was approved by the Danish Ethics Committees (application no. KA 94076m).

### The two-stage GWAS

The method is fully published by Antilla et al. A short description is given here to facilitate the understanding of our work.

In the discovery stage, a clinic-based sample of 3,279 migraineurs from Finland, Germany and The Netherlands was studied and genotyped at the Wellcome Trust Sanger Institute using Illumina (610K and 550K) SNP microarrays against 10,747 population-matched controls [10]. In the replication stage, a further 3,202 migraineurs from Iceland (using Illumina Human Hap 317K, 370K, 610K or 1M bead arrays at deCODE genetics), Denmark (using Centaurus platform [Nanogen inc.] at deCODE genetics), The Netherlands (TaqMan technology [Applied Biosystems, Life] at Leiden University Medical Center) and Germany (Illumina HumanHap 610K array at the Institute of Human Genetics at the Helmholtz Zentrum, Munich) were studied against 40,062 population-matched controls.

The diagnoses were given by headache experts using a combination of questionnaires and individual interviews based on the IHS 2004 criteria [11]. The following diagnostic subgroups were analyzed: (1) all migraine patients (‘all migraine’), (2) MA patients without attacks of MO (‘MA only’), (3) patients with attacks of both MA and MO (‘both MA and MO’), and (4) MO patients without attacks of MA (‘MO only’).

For the meta-analysis of discovery and replication samples the Cochran–Mantel–Haenszel (CMH) association analysis with a significance threshold of *p* ≤ 5 × 10^−8^ was used. In the discovery sample, 2,731 cases and 10,747 controls passed quality control steps, and 429,912 markers were successfully genotyped. Only one marker, rs1835740 on chromosome 8q22.1, showed significant association with migraine in the multi-population CMH analysis. The minor allele (A) of marker rs1835740 was associated with ‘all migraine’ with *p* = 5.38 × 10^−9^ and odds ratios ranging between 1.21 and 1.33. The result was confirmed in the replication study with a final *p* = 1.69 × 10^−11^ in the CMH meta-analysis for all migraine sample together.

In the HapMap Phase II data [14], marker rs1835740 is located between the two potentially interesting genes, MTDH and PCGP, which are both involved in glutamate homeostasis. The effect of the marker on gene expression was analyzed in human fibroblasts, primary T cells and lymphoblastoid cell lines obtained from umbilical cords. The risk allele A of rs1835740 was found to have significant correlation to higher MTDH expression in lymphoblastoid cell lines.

### Statistical methods

All data were processed and analyzed using Statistical Package for the Social Sciences (SPSS) version 18.0 in Windows 7.0. Two-tailed Student’s *t* test and one-way ANOVA was used to compare means for numerical data. *χ* ^2^ test was used to compare categorical data.

A comparison of carriers of the rs1835740 variant with non-carriers was performed. Thereafter carriers were subdivided into homo- and heterozygotes. No statistical tests were performed on homozygotes versus heterozygotes as the sample of homozygotes was too small. Finally, a sub-analysis in females was done. Sub-analysis in males was not performed as this sub-group was too small for a meaningful analysis.

## Results

136 out of 339 (40%) apparently non-related MO probands were carriers of the rs1835740 variant, hereof 20 out of 47 males and 116 out of 292 females. Of the carriers, 15 were homozygous (1 male and 14 females) and 121 were heterozygous (19 males and 102 females). In carriers, the average age was 43.8 ± 12.8, and in non-carriers 43.6 ± 11.5, *p* = 0.880. The average age of onset in carriers was 20.9 ± 12.0, and in non-carriers 20.9 ± 10.6, *p* = 0.995. The average duration of migraine was in the interval 4–23 h in 37.5% of carriers and in 36.9% of non-carriers, and 1–3 days in 54.4% of carriers and in 56.7% in non-carriers. Thus, carriers and non-carriers were comparable in average age, age of onset and average duration of migraine. Attack frequencies through life and during the past year were also evenly distributed for carriers and non-carriers (data not shown).

Table 1 summarizes the migraine-specific symptoms of carriers versus non-carriers with corresponding *p* values, and of homo- and heterozygotes, respectively. Table 2 describes the comorbidity. Provoking factors are described in Table 3, hormonal factors in females in Table 4. The effect and use of medicine is presented in Table 5.

**Table 1:** Symptoms associated with attacks of migraine in ‘migraine without aura’ patients divided in subgroups: carriers and non-carriers of the rs1835740 variant, and homo- and heterozygotes of the rs1835740 variant

Symptoms	Carriers	Non-carriers	p*	Homo	Hetero	Total number of answers
	% (*n*)	% (*n*)		% (*n*)	% (*n*)	
Unilateral location	83.9 (104)	87.7 (164)	0.338	80.0 (12)	84.4 (92)	311
Pulsating quality	81.0 (102)	82.6 (152)	0.710	71.4 (10)	82.1 (92)	310
Moderate or severe pain intensity	100.0 (136)	100.0 (203)	_	100.0 (15)	100.0 (121)	339
Aggravation by physical activity	91.0 (121)	93.0 (186)	0.500	80.0 (12)	92.4 (109)	333
Nausea	93.2 (124)	94.5 (188)	0.642	93.3 (14)	93.2 (110)	332
Vomiting	67.7 (86)	69.4 (127)	0.753	76.9 (10)	66.7 (76)	310
Photophobia	92.3 (120)	90.2 (175)	0.516	100.0 (14)	91.4 (106)	324
Phonophobia	88.4 (114)	82.7 (158)	0.165	100.0 (14)	87.0 (100)	320
Osmophobia	52.2 (59)	57.2 (97)	0.422	53.8 (7)	52.0 (52)	283

* p values for comparison of carriers and non-carriers

**Table 2:** Comorbidity in ‘migraine without aura’ patients divided in subgroups: carriers and non-carriers of the rs1835740 variant, and homo- and heterozygotes of the rs1835740 variant

Comorbidity	Carriers	Non-carriers	p*	Homo	Hetero	Total number of answers
	% (*n*)	% (*n*)		% (*n*)	% (*n*)	
Comotio	44.9 (61)	41.0 (82)	0.483	33.3 (5)	46.3 (56)	336
Cranial fracture	1.5 (2)	1.0 (2)	0.696	0.0 (0)	1.7 (2)	336
Encephalitis	0.0 (0)	0.5 (1)	0.409	0.0 (0)	0.0 (0)	336
Meningitis	1.5 (2)	2.0 (4)	0.719	6.7 (1)	0.8 (1)	336
Cerebral thrombosis	0.7 (1)	0.0 (0)	0.225	0.0 (0)	0.8 (1)	336
Cerebral hemorrhage	0.0 (0)	0.0 (0)	_	0.0 (0)	0.0 (0)	336
Transient cerebral ischemia (TCI)	0.0 (0)	0.0 (0)	_	0.0 (0)	0.0 (0)	336
Arterial hypertension	10.3 (14)	16.5 (33)	0.107	0.0 (0)	11.6 (14)	336
Tension type headache (TTH)	65.4 (89)	73.6 (147)	0.106	86.7 (13)	62.8 (76)	337

* p values for comparison of carriers and non-carriers

**Table 3:** Migraine provoking factors in ‘migraine without aura’ patients divided in subgroups: carriers and non-carriers of the rs1835740 variant, and homo- and heterozygotes of the rs1835740 variant

Provoking factors	Carriers	Non-carriers	p*	Homo	Hetero	Total number of answers
	% (*n*)	% (*n*)		% (*n*)	% (*n*)	
Attacks provokable	26.6 (29)	21.7 (26)	0.348	9.1 (1)	28.6 (28)	275
Physical activity	32.7 (37)	30.2 (51)	0.649	8.3 (1)	35.6 (36)	282
Stress	69.3 (79)	69.8 (120)	0.933	61.5 (8)	70.3 (71)	286
Weekend/holiday	49.6 (56)	51.5 (88)	0.753	53.8 (7)	49.0 (49)	284
Food	50.4 (57)	41.2 (70)	0.125	61.5 (8)	49.0 (49)	283
Alcohol	26.4 (29)	19.2 (32)	0.157	7.7 (1)	28.9 (28)	277
Hormonal factors, females only: menstrual migraine^a^	51.5 (50)	56.7 (85)	0.430	41.7 (5)	52.9 (45)	247

* p values for comparison of carriers and non-carriers

^a^Attacks associated to menstruation

**Table 4:** Effect of hormonal factors on migraine attack frequency, females only, in ‘migraine without aura’ patients divided in subgroups: carriers and non-carriers of the rs1835740 variant, and homo- and heterozygotes of the rs1835740 variant

Attack frequency	Unchanged	Higher	Lower	Irrelevant	Total number of answers
During pregnancy					252
Non-carriers	7.8 (12)	5.2 (8)	48.4 (74)	38.6 (59)	
Hetero	11.5 (10)	2.3 (2)	40.2 (35)	46.0 (40)	
Homo	0.0 (0)	0.0 (0)	41.7 (5)	58.3 (7)	
Using birth-control pills					246
Non-carriers	38.0 (57)	20.0 (30)	5.3 (8)	36.7 (55)	
Hetero	47.1 (40)	9.4 (8)	4.7 (4)	38.8 (33)	
Homo	36.4 (4)	9.1 (1)	9.1 (1)	45.5 (5)	
After meno pause					245
Non-carriers	10.8 (16)	7.4 (11)	6.8 (10)	75.0 (111)	
Hetero	15.3 (13)	10.6 (9)	2.4 (2)	71.8 (61)	
Homo	0.0 (0)	16.7 (2)	25.0 (3)	58.3 (7)

**Table 5:** Effect and use of medicine in ‘migraine without aura’ patients divided in subgroups: carriers and non-carriers of the rs1835740 variant, and homo- and heterozygotes of the rs1835740 variant

Effect and use of medicine	Carriers	Non-carriers	p*	Homo	Hetero	Total number of answers
	% (*n*)	% (*n*)		% (*n*)	% (*n*)	
Effect of triptans	83.2 (94)	85.8 (145)	0.601	84.6 (11)	83.0 (83)	282
Effect of prophylactic medication	33.0 (37)	28.5 (49)	0.582	46.2 (6)	31.3 (31)	284
Under current prophylactic medication	35.4 (40)	32.0 (54)	0.548	23.1 (3)	37.0 (37)	282
Other current daily medication	36.0 (41)	35.1 (60)	0.879	23.1 (3)	37.6 (38)	285
Previous/current treatment of med. overuse headache	32.1 (36)	29.2 (50)	0.604	23.1 (3)	33.3 (33)	283

* p values for comparison of carriers and non-carriers

No statistically significant association with the A-allele of rs1835740 was found in the comparison of carriers with non-carriers for any of the parameters studied. However, when carriers were divided in homo- and heterozygotes and compared to non-carriers, a tendency that heterozygotes were comparable to non-carriers, and that homozygotes tended to differ in a non-significant manner from these two other groups, could be observed.

A sub-analysis in females (not shown) gave the same results as in the whole material. No statistical calculations were done in males because of their small number. Numerically, males seemed to follow the general picture.

## Discussion

No statistically significant association with the A-allele rs1835740 was found for any of the parameters that were studied: symptoms, comorbidity, provoking factors or the effect and use of different medical treatment. However, while heterozygotes were comparable to non-carriers, homozygotes tended to differ in a non-significant manner in some parameters. Homozygosity for the A-allele of rs1835740 might thus have a small influence on the phenotype of MO, but it could easily be a false trend because of the small number of homozygotes. The sub-analysis in females did not show any difference from the overall analysis, and the male sample was too small for statistical calculation. Thus, the present study suggests that the rs1835740 variant has no influence on the clinical expression of MO.

### Why study MO as a separate entity?

It has been questioned whether MO and MA are two distinct disorders or a single entity [15, 16], but considerable evidence supports the former. All together, the different epidemiology, the different clinical features, difference in comorbidity, the presence or absence of measurable cortical spreading depression, different provoking factors, different effect of different preventive medicine and twin studies showing lower concordance-rate in MO than in MA, indicate that MO and MA are distinct disorders with some clinical and etiological overlap [5, 8, 17–20]. Therefore, the present study concentrated on MO only.

The original large study by Antilla et al. showed consistently stronger association with the presence of the rs1835740 A-allele for MA-only groups [10]. The present study indicates that the A-allele of rs1835740 probably has no crucial influence on the phenotype of MO. Maybe studies of clinical features in MA will reveal a more clear difference according to the presence or absence of rs1835740 A-allele in MA patients. This would further support the assumption that MO and MA are distinct disorders.

### Methodological considerations

In the absence of a biochemical marker or paraclinical test, the diagnosis of migraine is purely clinical. The differential diagnosis between tension-type headache (TTH) and migraine is often difficult, and also the differentiation between MO and MA is sometimes uncertain. We used an extensive, validated, semi-structured telephone interview performed by a physician or a trained senior medical student [12]. Thus, our clinical data are reliable as well as systematic and extensive.

The sample of MO patients in this study was recruited from a specialized clinical sample and thus represents a fairly severely affected group. We cannot exclude phenotypic differences between those with and without the variant in a population of less affected individuals.

The number of patients in this study was 339. This is usually an adequate number for phenotype–genotype correlation studies and sufficient for most purposes. Thousands of probands are needed to find a genetic association like the rs1835740 variant. However, if thousands of patients are needed to show significance of a difference of a certain clinical feature between two groups, this difference will not be of clinical importance. For analysis in relatively small groups, e.g., males, our material is insufficient. Most importantly, the group of homozygotes was far too small for a meaningful statistical analysis. It seems that if any phenotypical correlate of the variant exists, it must be found in this group. However, a very large material would be necessary to get enough homozygous patients.

### Migraine and glutamate

Marker rs1835740 is located between the two potentially interesting genes, MTDH and PCGP, which are both involved in glutamate homeostasis. The rs1835740 genotype was found by Antilla et al. to be significantly correlated to MTDH expression in lymphoblastoid cell lines [10]. In astrocytes, MTDH has been shown to downregulate GLT-1, the gene encoding the major glutamate transporter. The new genetic variant may thus contribute to the understanding of the role of glutamate in migraine. Glutamate is involved in central sensitization which is considered to be a crucial part of migraine pathophysiology [21], and glutamate accumulation increases the susceptibility to cortical spreading depression [22, 23].

Glutamate is the major excitatory neurotransmitter in the CNS and therefore plays a crucial role in the mediation of excitatory synaptic transmission [24]. The anatomic structures involved in the migraine pain pathway, including the trigeminal ganglion (TG), the trigemino-cervical complex (TCC) and thalamus, contain glutamate-positive neurons [25, 26]. Glutamate exhibits its actions through activation of ionotropic and metabotropic receptors (GluRs), and the pharmacological distinction of these is well documented [27]. Glutamate is released from the TCC in response to stimulation of dural structures. In TG neurons, glutamate is released along with calcitonin gene-related peptide (CGRP), and the majority of glutamatergic neurons in TG carry 5-HT_1B/D/F_ receptors, which could possibly modulate glutamate release [28]. Glutamate also plays a significant role in the transmission of nociceptive information in the sensory thalamus [29, 30].

Migraineurs have elevated levels of glutamate and glutamine in the cerebrospinal fluid compared with controls, and a positive correlation between glutamate levels and mean headache scores has been reported [31–33]. Although the variant only explains a small fraction of the overall genetic variance, it increases the interest in glutamatergic mechanisms in migraine.

### Personalized medicine and future perspectives

There is still an unmet need to find more effective, tolerable and safe treatments for migraine, especially regarding preventive agents. MO is clinically well defined as a syndrome, but it has not been possible to divide MO into subtypes based on clinical features. The tolerability, safety and efficacy of each type of preventive medicine are individual for every patient and not predictable. Thus, ‘trial and error’ is still the only possible treatment strategy. If a certain genotype was associated with certain clinical features, it would perhaps be possible to select patients for treatment based on clinical criteria. Unfortunately, the present study did not reveal a significant difference in clinical features according to the presence or absence of the rs1835740 variant. Another approach would be to compare the effect of drugs in patients with and without the variant. Especially, the effects of glutamate-modulating agents would be interesting to investigate. If this strategy gives results and as the technology of genotyping gets more economic and accessible, a future perspective could be to select patients for treatment based on genotyping [34].
